# Image-based high-throughput phenotyping enables genetic analyses of pod morphological traits in mungbean (*Vigna radiata* (L.) R. Wilczek)

**DOI:** 10.1093/g3journal/jkag106

**Published:** 2026-04-28

**Authors:** Venkata Naresh Boddepalli, Talukder Zaki Jubery, Steven B Cannon, Somak Dutta, Baskar Ganapathysubramanian, Arti Singh

**Affiliations:** Department of Agronomy, Iowa State University, Ames, IA 50011, United States; Department of Mechanical Engineering, Iowa State University, Ames, IA 50011, United States; Department of Agronomy, Iowa State University, Ames, IA 50011, United States; USDA-Agricultural Research Service, Corn Insects and Crop Genetics Research Unit, Ames, IA 50011, United States; Department of Statistics, Iowa State University, Ames, IA 50011, United States; Department of Mechanical Engineering, Iowa State University, Ames, IA 50011, United States; Department of Agronomy, Iowa State University, Ames, IA 50011, United States

**Keywords:** mungbean, GWAS, genomic prediction, image analysis, and high-throughput phenotyping

## Abstract

Mungbean (*Vigna radiata* (L.) R. Wilczek) is a vital source of digestible proteins and is well-suited for the plant-based protein industry. In this study, we analyzed pod morphological traits in the Iowa Mungbean Diversity (IMD) panel of 372 genotypes (2022–2023) using image-analysis-based phenotyping on 2,418 pod images. Pod morphological traits were extracted using deep learning image analysis, achieving excellent agreement with manual measurements (*r* > 0.96 for pod length (PL) and seed-per-pod (SPP)). Four complementary genome-wide association studies models identified 65 significant SNPs (−log_10_(*P*) ≥ 5.56) associated with pod curvature, length, width, and SPP traits. A significant SNP (5_35265704) on chromosome 4 was linked to pod dimensional traits, length, width, and curvature. A candidate gene, *Virad04G0076900*, located 15.6 kb from this SNP, is part of the GH3 gene family and has an *Arabidopsis* ortholog (*AT4G27260*) known for influencing organ elongation, pod, and seed development. Another SNP, 5_210437 on chromosome 6, has been found to be significantly associated with both PL and SPP. A candidate gene, *Virad06G0002400* (36.5 kb from this SNP), encodes a potassium transporter and shares homology with the *Arabidopsis* gene HAK5 (AT4G13420), known to influence pod growth. Image-based measurements achieved genomic prediction accuracies ranging from 0.61 to 0.85 across various traits, demonstrating comparable accuracy to manual methods for linear traits and up to 22% improvement for complex shape traits. These results highlight the potential of deep learning-assisted phenomics integrated with genomic tools to accelerate selection for improved pod architecture in mungbean breeding programs across the Midwestern United States and globally.

## Introduction

Mungbean (*Vigna radiata* (L.) R. Wilczek) is a warm-season food grain legume crop globally known for its high nutritional value. With over 7 million hectares of global cultivated area, predominantly grown in Asian countries, followed by Australia, several African nations, South America, and parts of the USA (Kohno et al. 2018; Nair and Schreinemachers 2020; Somta et al. 2022; Fumia et al. 2023). Mungbean seeds contain 20%–30% digestible proteins, as well as dietary fiber, essential amino acids, iron, and folates (Nair and Schreinemachers 2020; Sandhu and Singh 2021; Somta et al. 2022). Mungbean has historically been a staple protein source in South and Southeast Asia (Somta et al. 2022). In recent years, mungbean has emerged as a promising option for crop diversification in North America, including the United States and Canada, where demand for sustainable plant-based protein sources is growing (Sandhu and Singh 2021). Its ability to thrive in hot, drought-prone conditions, with a short lifecycle (60–75 d after planting), to share the same infrastructure used for soybean cultivation with minimal adjustments, makes it a valuable addition to existing cropping systems. Despite its global importance, mungbean remains underutilized and underrepresented in U.S. agricultural systems and breeding programs (Sandhu and Singh 2021). However, recent initiatives, such as the establishment of the Iowa Mungbean Diversity (IMD) panel, aim to address this gap by evaluating germplasm under the U.S. field conditions for agronomic suitability (Sandhu and Singh 2021). These efforts highlight the potential of mung beans not only to supplement domestic protein needs but also to enhance ecological and economic resilience through diversification.

In legumes, pods are essential reproductive structures that can directly affect yield through their morphological traits, including pod length (PL), width, curvature, and the number of SPP. These traits are potential determinants of harvest index and yield, also affecting threshability, seed uniformity, and market value (Funatsuki et al. 2014; Di Vittori et al. 2021; Li et al. 2024; Njau et al. 2024). During mungbean domestication, pod architecture and dehiscence were important traits, with favorable selection toward larger, nonshattering pods that contain more SPP (Li et al. 2024). Previous studies documented a positive correlation between PL and the number of SPP with seed yield and 100-seed weight in mungbean (Liu et al. 2022; Chang et al. 2023; Manjunatha et al. 2023; Li et al. 2024), common bean (García-Fernández et al. 2021), cowpea (Xu et al. 2017), soybean (Chen et al. 2023), and faba bean (Li and Yang 2014). These studies highlight the need to understand the genetic architecture underlying these yield-contributing traits, where genome-wide association studies (GWAS) and comparative mapping enable the identification of major loci and candidate genes; however, there have been limited studies on mungbean pod morphological traits using large diversity panels and multienvironment phenotypic data (Liu et al. 2022; Somta et al. 2022; Manjunatha et al. 2023; Li et al. 2024). The complex quantitative nature of pod morphological traits combined with their high heritability and breeding importance, makes them ideal candidates for genomic dissection using modern molecular approaches. However, understanding the genetic control of these traits is essential for marker-assisted selections that can accelerate breeding progress and enable precise manipulation of pod architecture in mung bean improvement programs.

Recent advancements in genomics have led to increased access to modern genetic tools, such as GWAS, comparative mapping, and genomic prediction (GP), for accelerating trait discovery and new cultivar development (Han et al. 2022; Somta et al. 2022; Fumia et al. 2023; Ahmed et al. 2024; Chiteri et al. 2024). GWAS has effectively identified quantitative trait loci (QTL) related to important agronomic traits in mungbean, such as 100-seed weight, flowering time, leaf and root morphology, pod dehiscence, biotic and abiotic resistance, using the high-density single-nucleotide polymorphism (SNP) data from various mung bean panels (Chiteri et al. 2022, 2023, 2024; Han et al. 2022; Manjunatha et al. 2023; Ahmed et al. 2024; Kohli et al. 2024; Li et al. 2024; Iqbal et al. 2025). Comparative mapping with closely related legumes, such as cowpea (*Vigna unguiculata* (L.) Walp.), common bean (*Phaseolus vulgaris* L.), and soybean (*Glycine max* (L.) Merr.), has revealed conserved synteny and orthologous regions that regulate domestication traits. This enables researchers to understand gene functions across different species (Manjunatha et al. 2023; Chiteri et al. 2024). Moreover, GP models that include trait variations have shown great potential for increasing selection accuracy and reducing breeding cycles (Miller et al. 2023; Tang et al. 2024; Escamilla et al. 2025; Mbebi et al. 2025). While GP has been extensively implemented in major legume crops such as soybean for over a decade (Keller et al. 2020; Shao et al. 2022; Huynh et al. 2024; Van der Laan et al. 2025), its application in mungbean remains limited. To the best of our knowledge, no GP studies have been published specifically for pod morphological traits in mungbean. By integrating phenotypic data from multiple environments with genome-wide marker effects, these models provide a scalable approach to enhance genetic improvement in mung bean. However, precise phenotyping of pod morphological traits for genetic studies is a significant challenge in legumes. Traditional manual measurements are labor-intensive, time-consuming, and prone to inter-rater and intrarater variability. This variability can diminish the statistical power of subsequent GWAS and GP models (Chang et al. 2021; Li et al. 2021; Gill et al. 2022; Yang et al. 2024; Liu et al. 2025).

To address these challenges in mungbean breeding, this study integrated high-throughput image analysis with genomic approaches to examine the genetic structure of pod morphological traits. Our study objectives were to: (i) develop and validate machine learning model for the automated extraction of PL, width, curvature, and SPP traits from scanned images; (ii) characterize the phenotypic diversity and trait relationships within the IMD panel; (iii) identify the marker-trait association of these traits through genome-wide association studies; and (iv) evaluate the effectiveness of image-based phenotyping compared to manual phenotyping for GP. This integrated approach provides essential tools and genetic insights aimed to accelerate mungbean improvement through marker-assisted breeding and genomic selection.

## Materials and methods

### IMD panel and experimental design

The IMD panel initially comprised 482 accessions. For this study, we utilized a filtered core set of 372 genetically unique accessions, as previously characterized by (Chiteri et al. 2022) using identity-by-state similarity and Nei's genetic distance to eliminate redundant duplicates and accessions with poor genotypic quality. Among the 9 check varieties included in the study, 4 elite lines (AVMU 0001, AVMU 0201, AVMU 8501, and AVMU 9701) were sourced from the World Vegetable Center, while the remaining 5 were commercial cultivars. The IMDP panel was tested in the experimental fields of Iowa State University's Agricultural Engineering and Agronomy in Boone, Iowa (coordinates: 42°00′58.5″N, 93°46′16.7″W). In 2022, the initial panel with 481 genotypes was planted on June 1; in 2023, the IMD panel with 372 genotypes was planted on June 2 at the Burkey and Bruner farms. One hundred seeds of each genotype were planted in 3-row plots in a randomized complete block design with 2 blocks per location. A spacing of 2 inches between plants and 15 inches between rows was utilized. Standardized agronomic practices were implemented during this experiment, including a rain-based irrigation system.

### Image acquisition

**Fig. 1.**
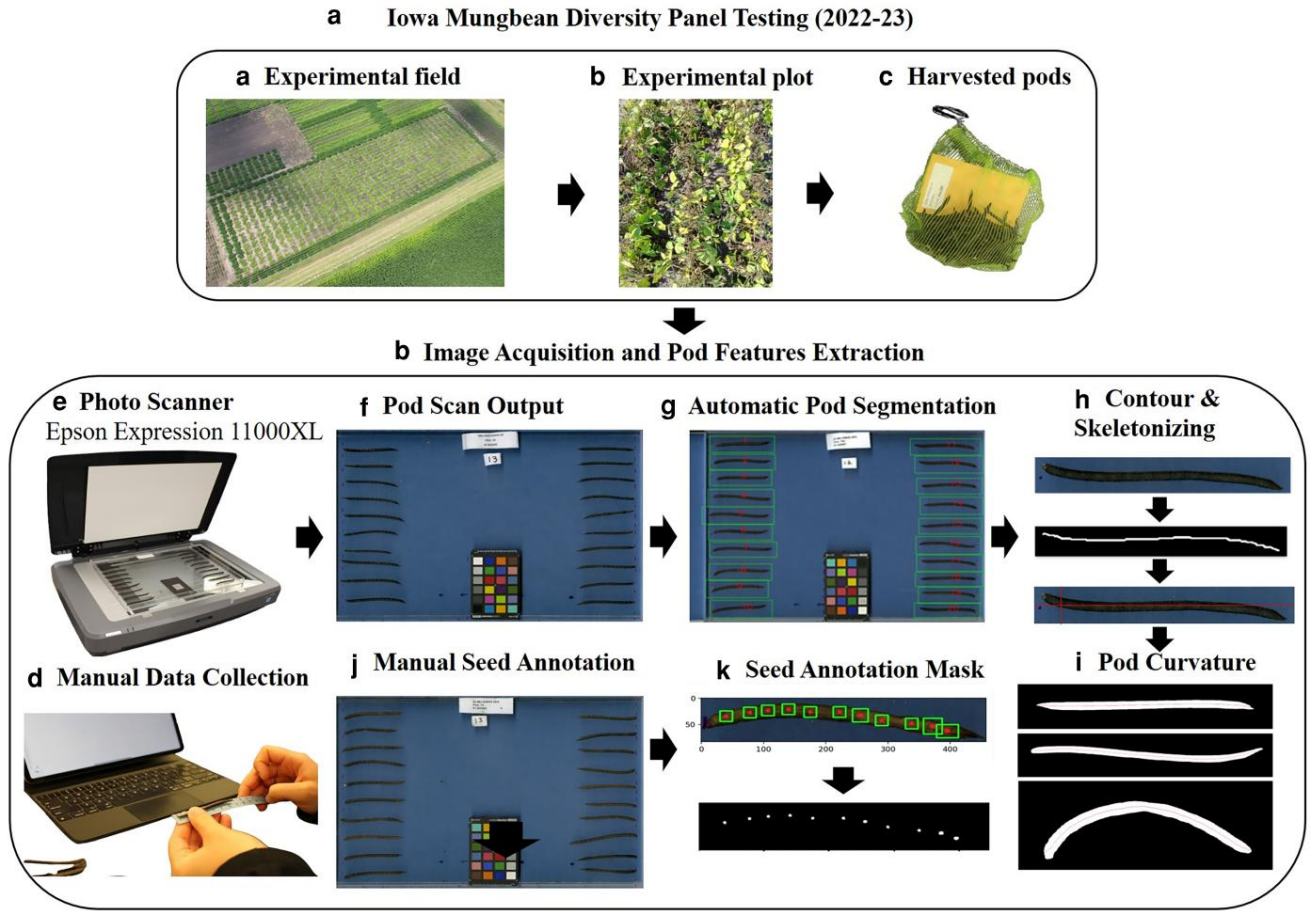
Flowchart showing the workflow for image-based mungbean pod trait extraction in the Iowa mungbean diversity panel testing (2022–2023). a) Experimental field, matured plots, and harvested pods. b) Image acquisition and feature extraction pipeline.

All genotypes were harvested at the R6 stage when 85%–90% of the pods had matured on the plot. The clusters with mature pods at the top 3 nodes of the plants in the middle row of 3 rows were cut tags with scissors, bagged in a nylon mesh bag along with the plot identifier tags, and stored in the lab for image acquisition and manual data collection. An Epson Expression 11,000 XL photographic scanner was used to capture RGB images of 20 randomly selected pods from each genotype at 300 dots per inch (dpi). The randomly selected 20 pods were arranged as shown in Fig. 1f on a transparent plastic tray, 10 pods each to the right and left sides, and the color calibration chart in the lower center of the tray, and the plot identifier label with trial name, plot ID, and genotype ID in the top center facing inward position. Images were acquired at a spatial resolution of 300 dpi, resulting in dimensions of 4299 × 3,035 pixels (corresponding to a physical field of view of ∼14.3 × 10.1 inches). These components were consistently placed at the top center to enable deterministic cropping. A uniform blue background was used to aid segmentation, though lighting artifacts from the glass slide introduced complexity.

### Manual data collection and seed annotation

PL and SPP data were manually recorded to test the model accuracy. For pod curvature (PC), the categories: (i) least curved, (ii) slightly curved, and (iii) curved, were utilized for manual phenotyping. PL was measured from the pedicle-pod joint to the tip of the pod beak using flexible centimeter scales on harvested pods from 2 environments in 2023. The SPP was counted without splitting the pods harvested from the 2022 to 2023 experimental plots. To train the model to extract SPPs, 1,678 images from each environment in 2022 and 2023 were annotated from a total of 2,418 scanned images, while images from the third location were used as the test set. The scanned images were opened in Microsoft Paint (Windows 10), and the left-side 10 pods were annotated with red dots at the seed position. The data was recorded in an Excel sheet for further analysis.

### Image analysis and trait extraction

We developed an automated image-analysis pipeline to extract morphological and SPP traits from scanned images of 20 mungbean pods. The workflow comprises: (i) OCR-based metadata extraction, (ii) pod detection and cropping, (iii) background removal, (iv) trait extraction (PL, PW, and PC), and (v) seed count estimation (SPP). Figure 1 illustrates the complete image-analysis pipeline.

### Metadata tag identification

Tags were consistently located in the top-middle region for pod images and the top-middle of each Petri plate. We binarized the cropped region and identified the largest connected component as the tag. OCR (Tesseract v5.3.0) was used with a restricted character set to match expected formats (eg Plot: 1234, PI56789). Outputs were validated with regular expressions, and cropped tags were saved for traceability.

### Pod detection, cropping, and background removal

Pod segmentation was achieved using a U-Net model with a ResNet34 encoder, trained using annotated data via LabelMe. The 20 largest components were sorted from bottom-left to top-right to standardize indexing. Bounding boxes were extracted to crop individual pods (Fig. 1g). Each cropped pod was further processed using a DeepLabV3 model (ResNet34 backbone) for pod-background segmentation. The largest connected component was retained and refined with morphological operations to eliminate edge noise. We produced 3 outputs per pod: a clean binary mask, a visual overlay, and a trait-ready mask (Shen et al. 2020; Wang et al. 2022; Dhiyanesh et al. 2025; Zhou et al. 2025).

### Pod trait extraction: length, width, curvature, and SPP

Morphological features were extracted as follows: (i) Calibration and pixel-to-physical conversion: A calibration standard (ruler or calibration card) was included in each scan to establish the pixel-to-centimeter conversion factor. The conversion factor of 1 px = 0.00847 cm was determined by measuring known distances on the calibration standard across multiple scans (*n* = 50) and averaging the ratios. This corresponds to the theoretical resolution of 300 dpi used by the Epson Expression scanner (1 inch = 2.54 cm; 300 pixels/inch = 0.00847 cm/pixel). The calibration was validated by measuring objects of known dimensions, achieving a measurement accuracy of ± 0.1 mm. (ii) Skeletonization: We inverted the binary mask and traced the midline by computing the midpoint between the topmost and bottommost contours for each x-coordinate. (iii) PL: The midline's pixel length was converted to centimeters using the validated conversion factor. (iv) PW: 100 midline points were sampled, and the width was estimated as twice the shortest Euclidean distance from each midline point to the outer contour. We computed mean, max, and median widths. (v) PC (straightness index): A regression line fits midline coordinates; RMSE was used as a curvature metric. (vi) SPP estimation: A U-Net (ResNet34 encoder) was trained to segment seeds. To address noise and variability, we used an ensemble of 5 checkpoints (epochs 5–9) and averaged their outputs. Thresholded segmentations were used to count connected components, which served as seeds. Ensemble averaging improved robustness, though some failure cases remain.

### Implementation and reproducibility

The pipeline was implemented in Python 3.10 with PyTorch 1.13. Segmentation models were accessed via the segmentation models from PyTorch package. Preprocessing was handled using OpenCV (Bradski 2000) and Albumentations (Buslaev et al. 2020). Outputs are saved as CSV files. All models ran on NVIDIA GPUs.

### Descriptive statistical analysis of pod traits

Data from 20 random pods per genotype were averaged for each genotype prior to modeling (Supplementary Table 1). The normality of the phenotypic distribution for each trait was assessed using the Shapiro–Wilk test (W). Descriptive statistics, including standard deviation (SD) and coefficient of variation (CV), were calculated to characterize the data distribution. Outliers were identified and removed using the mean ±3 SD method, except for manual PC (scored 1–3), for which no outliers were removed to preserve categorical variation. For continuous traits demonstrating significant deviation from normality (*P* < 0.05), a Box–Cox power transformation was applied to normalize the distributions (Supplementary Table 2), ensuring the assumptions of the linear mixed model were met.

Phenotypic data were analyzed using a linear mixed model (LMM) implemented in the lme4 package in R (Bates et al. 2015) within the multienvironment trial framework described by (Piepho et al. 2003). The model is specified as follows:


$$Y_{ijk}=\mu +g_{i}+\alpha_{j}+\beta_{k(j)}+(g\alpha {)}_{ij}+\varepsilon_{ijk}$$

where *Y_ijk_* is the observed phenotype; *µ* is the overall mean; *g_i_* represents the genotype effect; *α_j_* is the environment effect (defined as year-location combinations); *β_k(j)_* is the random block effect nested within the environment, distributed as *β* ∼ *N*(0, $\sigma_{\beta }^{2}$); (*gα*)*_ij_* is the genotype × environment interaction; and *ε_ijk_* is the residual error distributed as *ε* ∼ *N*(0, $\sigma_{e}^{2}$).

To generate GWAS phenotypes, best linear unbiased estimators (BLUEs) were calculated by treating genotypes and environments as fixed effects (Supplementary Table 3). Conversely, for the estimation of variance components, genotypes were treated as random effects assuming independence (*g ∼ N* (0, $\;I\sigma_{g\alpha }^{2}$)). This independence assumption was selected to estimate total genetic variance (broad sense) without the shrinkage imposed by a kinship matrix (*K*) at this stage.

Broad-sense heritability (*H*^2^) was estimated using the method proposed by (Cullis et al. 2006), which is robust for unbalanced data in multienvironment trials:


$$H_{\mathrm{Cullis}}^{2}=1\;-\;\frac{v_{\Delta }^{\mathrm{BLUP}}}{2\sigma_{g}^{2}}$$

where $v_{\Delta \ldots }^{\mathrm{BLUP}}$ represents the mean variance of a difference between 2 BLUPs for the genotypic effect, and $\sigma_{g}^{2}$ denotes the genotypic variance component. We performed all analyses using the open-source R software (R Core Team 2021).

### Linkage-disequilibrium analysis and population structure analysis

Genome-wide linkage disequilibrium (LD) was assessed using TASSEL v5.0 (Bradbury et al. 2007) based on a genotype set of 31,058 SNPs obtained after alignment to the mung bean telomere-to-telomere (T2T) reference “*Weilv*-9' genome assembly (Jia et al. 2025). To ensure robust estimates, the dataset was filtered to retain only variants with a minor allele frequency (MAF) > 0.05 and missing data <10%, resulting in a final set of 17,928 high-quality SNPs. Pairwise LD was estimated using the squared correlation coefficient (*r*^2^) for all SNP pairs within a sliding window of 50 SNPs. LD decay was modeled and visualized using a custom Python script. To reduce noise and improve model fitting, SNP pairs were grouped into distance bins based on quantiles, and the mean *r*^2^ was calculated for each bin. A nonlinear regression model based on (Hill and Weir 1988) was fitted to these binned means using the equation $r^{2}=\;\frac{1}{1+pd}+c$, where *d* is the physical distance in kb, *P* is the population recombination parameter, and *c* is the background LD. The model fitting was weighted by the inverse standard error of the mean for each bin. The LD decay distance was defined as the physical distance at which the fitted curve intersected the critical threshold of *r^2^* = 0.2. All statistical fitting and plotting were performed using the *scipy.optimize* and *matplotlib* libraries in Python.

To asses population structure, an Identity-by-descent (IBD)-based genetic relatedness among all accessions was calculated using the GAPIT R package (Lipka et al. 2012), and hierarchical clustering was performed using Ward's method with squared Euclidean distances (Szekely and Rizzo 2005). The resulting dendrogram and pairwise relatedness heatmap were visualized to assess population groupings. Principal component analysis (PCA) was conducted in TASSEL v5.0 to examine genetic relationships among accessions and quantify the variance explained by the major axes of genetic variation. PCA results were visualized with samples colored by their geographic origin based on passport data. All statistical analysis and visualization were performed using R, Python, and TASSEL v5.0.

### Phenotypic PCA

Phenotypic PCA was conducted to examine the phenotypic structure and relationships among various pod morphological traits. Pod morphological traits (image and manual data) were analyzed using the BLUEs from 372 genotypes to ensure consistency for subsequent genetic association analyses. All trait values were standardized (mean = 0 and standard deviation = 1) to ensure each trait was given equal weight, regardless of its measurement scale. The BLUEs data contained no missing values. PCA was executed using singular value decomposition to extract the principal components, with the number of components retained based on eigenvalues >1 and the cumulative variance explained. The contribution of each trait to the principal components was assessed based on the component loading calculations. The analysis was carried out using the *scikit-learn* package in Python (version 1.3.0) (Pedregosa et al. 2011). The variance explained by each component, and the cumulative variance were calculated to evaluate the dimensionality of phenotypic variation. A biplot visualization was created to display both genotype scores and trait loadings within the principal component space.

### Genotyping

Raw genotyping-by-sequencing data from the IMD panel (Sandhu and Singh 2021) were aligned to the gap-free, telomere-to-telomere (T2T) *V*. *radiata* reference genome assembly *Weilv-9* (Jia et al. 2025). This T2T assembly was utilized to resolve large-scale structural variations and chromosomal translocations present in prior fragmented assemblies (VC1973A). Following alignment, variant calling was performed, and the genotype file was filtered to include only the 372 genotypes selected for this study (Chiteri et al. 2023), resulting in 31,058 SNPs. After further quality control, removing markers with >15% missing data and a MAF < 0.05, the final high-quality genotypic dataset comprised 17,928 SNPs distributed across the 11 chromosomes of the *Weilv-9* assembly.

### Genome-wide association study

To dissect the genetic architecture of these traits, we employed a comprehensive multimodel approach using the GAPIT v3 package (Wang and Zhang 2021), utilizing BLUEs as the phenotypic input. We utilized a single-locus mixed linear model (MLM) (Zhang et al. 2010) and 3 multilocus models: Fixed and random model circulating probability unification (FarmCPU) (Liu et al. 2016), Bayesian information and linkage-disequilibrium iteratively nested keyway (BLINK) (Huang et al. 2019), and Selection of Variables with Embedded screening using Bayesian methods (SVEN) (Li et al. 2023) for GWAS analysis.

To account for confounding effects due to population structure and relatedness, both PCA and a Kinship matrix were incorporated into the models. The Kinship matrix (*K*) was estimated using the (VanRaden 2008) method based on the full set of 17,928 SNPs to model the random polygenic effect. Simultaneously, the first 3 principal components (PCs) were included as fixed effect covariates (Q matrix) to correct for stratification. This specific partitioning of fixed (PCs) and random (Kinship) effects ensures robust control of false positives without over-correcting for true biological signals. Genome-wide significance was determined using a Bonferroni-corrected threshold of −log_10_(*P*) ≥ −log_10_ (0.05/*n*), where *n* is the total number of markers (*n* = 17,928). This corresponded to a significance threshold of *P* < 3.72 × 10–6 or −log10(*P*) ≥ 5.56. Visualization of GWAS results from BLINK, FarmCPU and MLM, including Manhattan and Q-Q plots, was performed using the “qqman” (Turner, 2014) and “ggplot2' packages in R. The SVEN method, which uses an embedded screening approach for variable selection, outputs phenotypic variance explained (PVE) for selected loci rather than continuous probability values and was therefore reported only in tabular summaries (Supplementary Table 4). The PVE for each significant marker-trait association was estimated using the coefficient of determination (*R*^2^) derived from a linear regression of the phenotype on the marker genotype, as implemented in the *GAPIT*, and *BRAVO* (SVEN) packages.

### Candidate gene identification and comparative mapping

Two common SNPs associated with pod morphological traits were identified for downstream analysis. Candidate gene searches were performed using the legume information system (LIS) JBrowse tools (https://www.legumeinfo.org/) to identify genes located within a 223 kb region around the significant SNP. This analysis was based on the average LD decay observed in the population. To systematically prioritize high-confidence candidates from these broader regions, we employed a step-wise filtering approach. First, the identified protein sequences were compared using BLASTP (Wheeler and Bhagwat 2008) with the protein databases of soybean (*Glycine max* (L) Merr), cowpea (*Vigna unguiculata* (L.) Walp.), and common bean (*P*. *vulgaris* L.) available in the legume information system and with *Arabidopsis thaliana* in the *Arabidopsis* Information Resource (TAIR-https://www.arabidopsis.org/tools/blast/) using an E-value cutoff of 1×10^−20^. Next, these orthologous matches in *Arabidopsis* and other legumes were systematically evaluated for gene ontology and functional descriptions specifically related to relevant physiological processes, such as cell elongation, phytohormone signaling, and sink-source dynamics. A chord diagram to visualize synteny between mungbean, soybean, cowpea, and common bean proteins was generated on the SequenceServer (version 3.1.2) using BLASTP 2.15.0 (Priyam et al. 2019) in LIS (https://sequenceserver.legumeinfo.org/). Additional comparative mapping tools available on the LIS: ZZBrowse, JBrowse, Genome Context Viewer, Gene Family Search (Dash et al. 2016; Redsun et al. 2022) were used for comparative genomic analysis.

### Genomic prediction

To evaluate the accuracy of GP for pod morphological traits, a ridge regression best linear unbiased prediction (rrBLUP) model was employed using the rrBLUP package (Endelman 2011). GPs were conducted using a marker matrix derived from 17,928 SNP markers and phenotypic values collected through both manual and image-based phenotyping methods. To maximize the additive genetic variance available for prediction, the model relied on the realized genomic relationship (kinship) matrix to account for population structure and cryptic relatedness. Principal components from the PCA were not included as fixed effects, as the kinship-based rrBLUP framework sufficiently controls for stratification without over-parameterizing the model. For each trait, we performed 10-fold cross-validation (CV), which balances bias and variance in prediction accuracy estimates and maintains adequate training set size for our population of 372 genotypes. The phenotype values (BLUPs) (Supplementary Table 5) for each genotype were across environments and randomly divided into 10 equal-sized subsets, ensuring balanced representation across the diversity panel. In each iteration, 9 subsets (90%) were used to train the model, while the remaining subset (10%) was used to test prediction accuracy. This process was repeated 10 times, so each sample was used once for validation. Genomic prediction accuracy (GPA) was evaluated using the Pearson correlation coefficient, coefficient of determination (*R*^2^), mean squared error, and prediction bias between observed and predicted phenotypes. In each CV-fold, the Pearson correlation coefficient was calculated between the genomic estimated breeding values (GEBVs) (Supplementary Table 6) predicted by the rrBLUP model and the observed trait BLUPs for each genotype in the test set. In each fold, a MAF filter (≥ 0.05) was applied to remove rare SNPs that could cause overfitting, and markers with zero variance within the training set for each fold were excluded to maintain numerical stability. Performance metrics were calculated per fold, averaged across 10-folds, and standard errors were computed to assess prediction reliability. To enable robust comparison between image-based and manual phenotyping approaches, identical CV partitions were used within each trait. A selection coincidence analysis was also conducted to assess agreement between phenotypic and GP. For each trait, the top twenty genotypes based on both phenotypic BLUPs and GEBVs were identified, and the selection coincidence index (SCI) was calculated based on the proportion of the genotypes common to both top-20 lists (coincidence = number of common genotypes/20).

## Results

### Image analysis and trait extraction accuracy

**Fig. 2.**
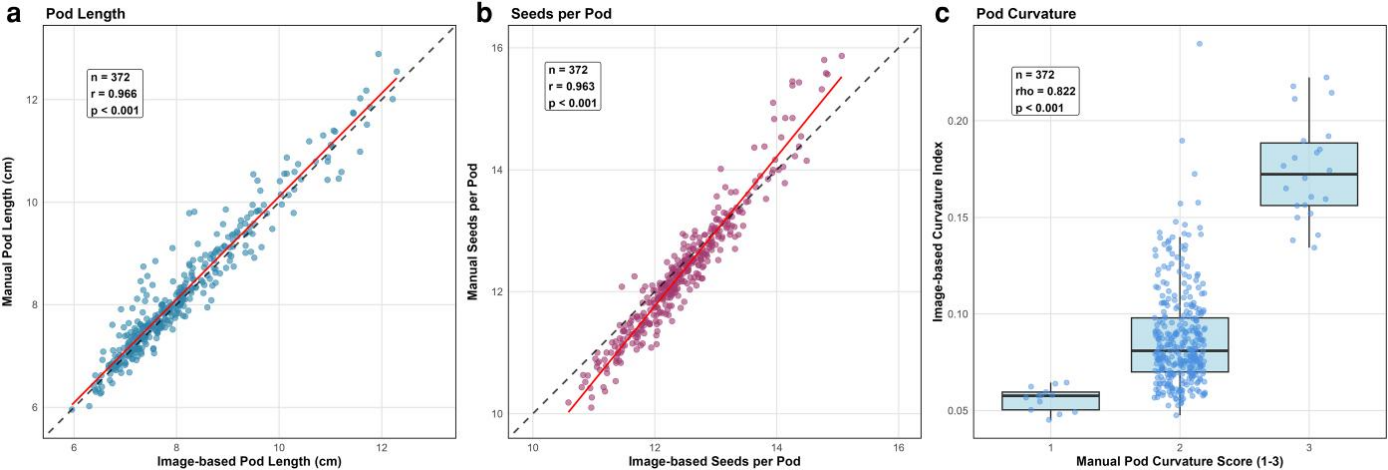
Comparison of manual vs image-based phenotyping methods. a) Linear regression of PL (cm). b) Linear regression of SPP. c) Boxplot comparison of manual PC scores (1–3 scale) vs image-derived curvature indices.The solid line in (A) and (B) represents the fitted regression, and the dashed line indicates a 1:1 relationship.

Our machine learning-based analysis model showed high accuracy in quantifying the pod morphological traits. The PL measurements (*r* = 0.96, *P* < 0.001) and SPP (*r* = 0.963, *P* < 0.001) showed exceptionally high agreement between image-based and manual measurement methods (Fig. 2a and b). Similarly, the image-derived curvature index showed a strong rank correlation (rho = 0.82) with manual scores, validating the high-throughput method (Fig. 2c). This demonstrates the model accuracy and confirms the reliability of the automated phenotyping approach. Since manual measurements for PW were impractical, we relied solely on the model for this data in our further analysis.

### Phenotypic variation and trait relationships

**Fig. 3.**
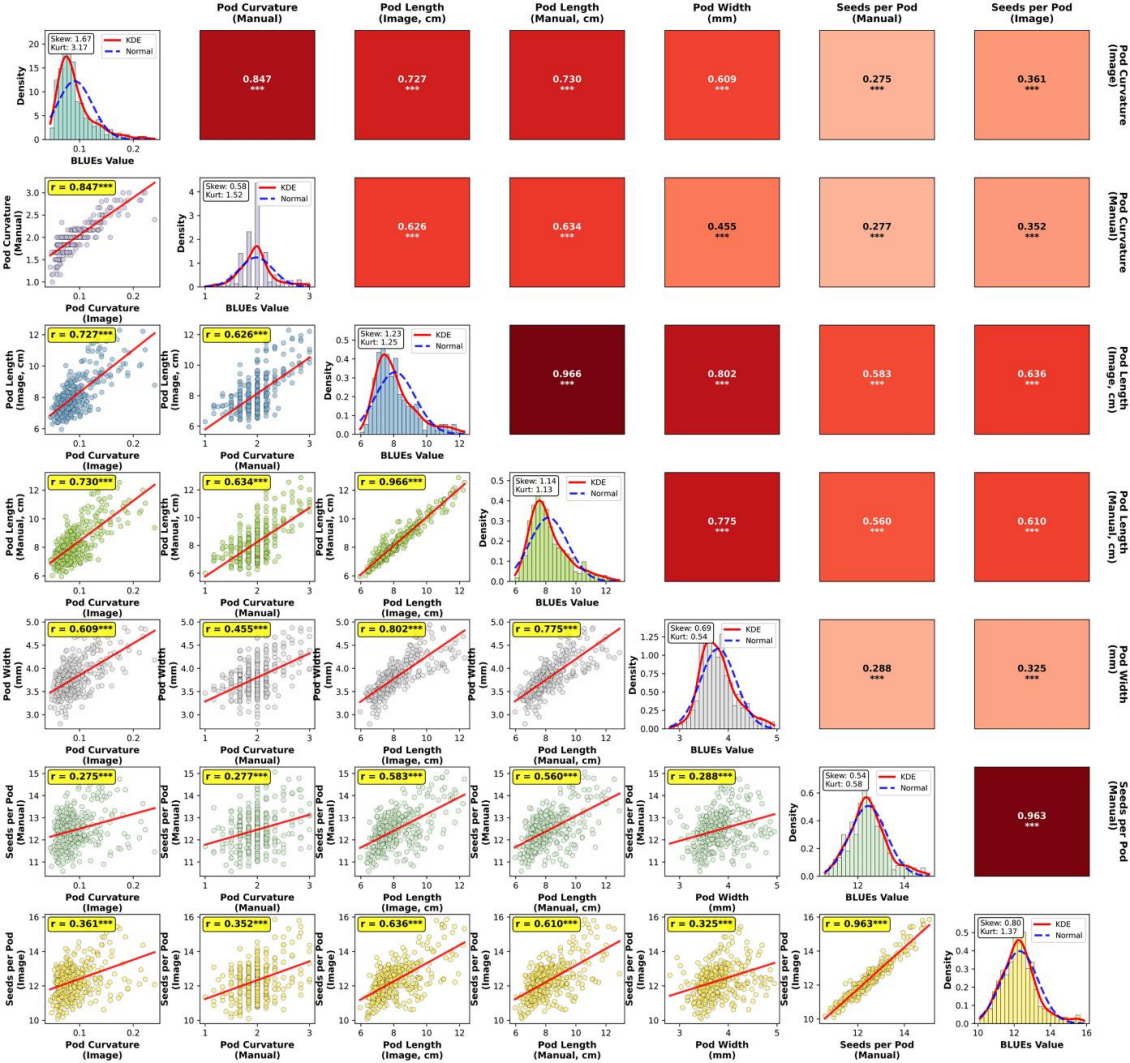
Correlation matrix and phenotypic distributions of pod morphological traits. Heatmap showing pairwise Pearson correlation coefficients among manual and image-based traits. Stronger correlations are indicated by darker shading. Histograms showing the distribution of individual traits.

A comprehensive phenotypic evaluation of pod morphological traits, based on both image analysis and manual measurements, revealed significant variation among the IMD panel accessions (*P* < 0.001; Table 1 and Fig. 3). Genotypic variance (*V_g_*) consistently exceeded environmental variance (*V_e_*) and genotype × environment interaction variance (*V*_ge_) for all image-derived traits. Broad-sense heritability (*H*^2^) for image-based measurements ranged from 0.74 to 0.91. For PL, the image-based heritability (*H*^2^ = 0.91) was higher than that of manual measurements (*H*^2^ = 0.81). An image-based analysis of PC yielded an *H*^2^ of 0.83 compared with 0.55 for manual scoring. Heritability estimates for SPP were comparable between manual (*H*^2^ = 0.75) and image-based (*H*^2^ = 0.74) methods. The coefficient of variation (CV%) was highest for PC (35%) and lowest for SPP (6%).

**Table 1.** Descriptive statistics, variance components, and broad-sense heritability (*H*^2^) estimates for pod morphological traits assessed by manual and image-based phenotyping in 372 mungbean genotypes.

Trait	Method	Mean	Range	SD	CV%	V_g_	V_e_	V_ge_	*H* ^2^
PL (cm)	Manual	8.19	5.96–12.89	1.26	15.3	1.25	0.2	0.05	0.81
	Image	8.09	5.96–12.29	1.21	14.9	1.3	0.1	0.02	0.91
Pod width (mm)	Image	3.79	2.80–4.94	0.37	9.7	0.12	0.01	0	0.91
PC	Manual (1–3 scale)	1.98	1.00–3.00	0.7	35.3	0.25	0.15	0.05	0.55
	Image (index)	0.09	0.05–0.24	0.03	35	0.01	0	0	0.83
SPP	Manual	12.45	10.58–15.07	0.8	6	0.55	0.15	0.05	0.75
	Image	12.31	10.10–15.87	0.9	8	0.6	0.18	0.04	0.74

Phenotypic correlation analysis revealed highly significant positive associations (*P* < 0.001) among pod dimensions (Fig. 3). PL and width exhibited strong correlations, ranging from *r* = 0.77 to *r* = 0.80. Image-derived PC was moderately associated with PW (*r* = 0.61) and PL (*r* = 0.73), whereas manual curvature scores displayed weaker correlations with PW (*r* = 0.45). SPP showed moderate positive associations with PL (*r* = 0.56–0.63) but weak correlations with PC and PW (*r* = 0.27–0.36).

### LD analysis and population structure analysis

**Fig. 4.**
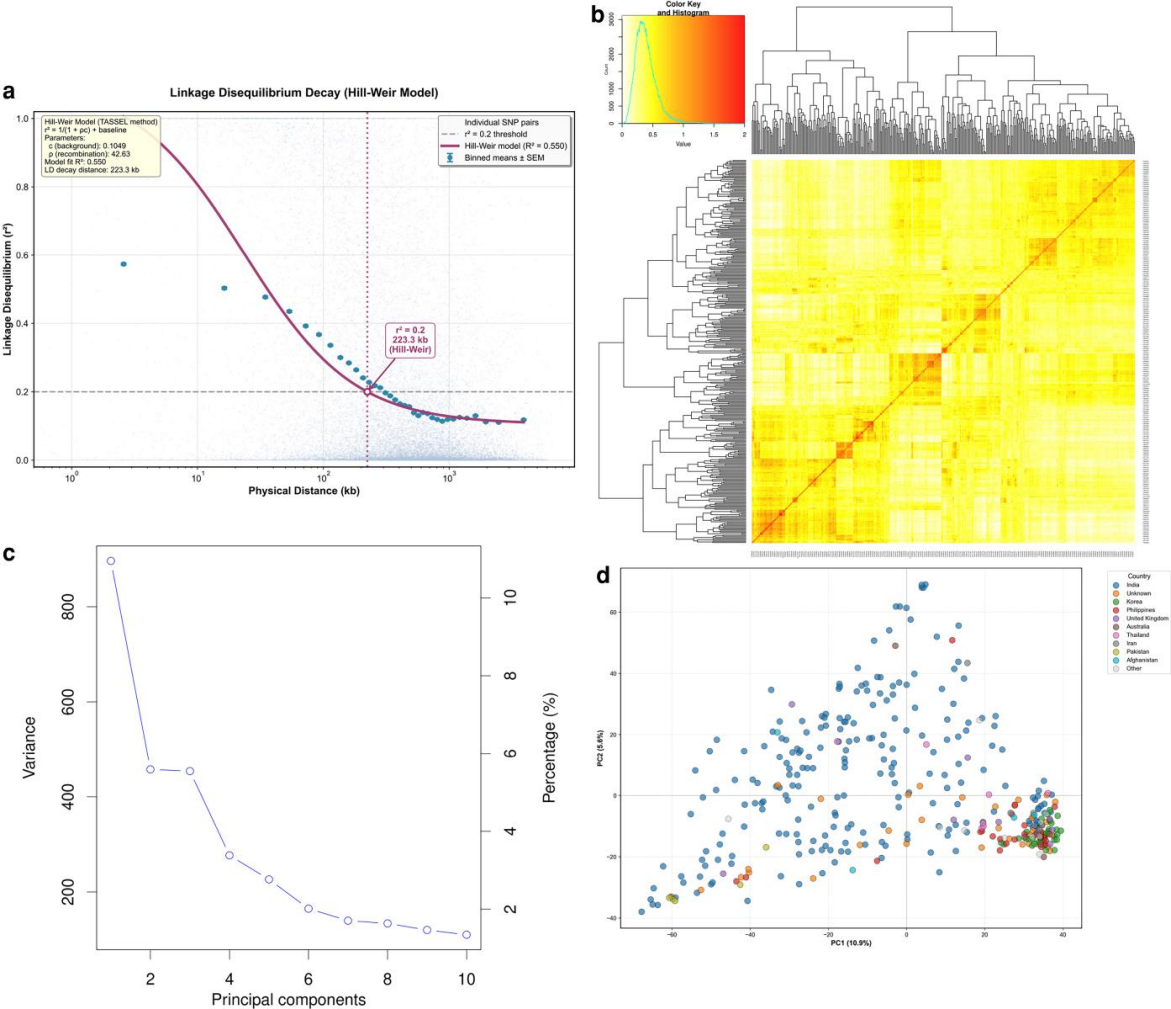
LD analysis and population structure of the Iowa mung bean diversity panel. a) LD decay curve showing *r*^2^ values against physical distance. b) Bar plot of population structure at *K* = 4, with individuals partitioned into 4 clusters. c) Delta K plot supporting *K* = 4 as the optimal number of subpopulations. d) PCA plot showing the percentage of variation explained by the first 2 principal components.

LD was estimated for 17,928 SNP pairs mapped to 11 mung bean chromosomes. Figure 4a illustrates the LD decay plot. At ∼223.3 kb of physical distance, the LD dropped below the threshold of *r*^2^ = 0.2.

Population structure analysis based on IBD and hierarchical clustering revealed a clear genetic stratification within the IMD panel (Fig. 4b). The dendrogram and associated heatmap showed distinct clustering patterns, with samples grouping primarily by geographic origin. PCA further confirmed the existence of structured genetic variation (Fig. 4d), with the first 2 principal components (PC1 and PC2) accounting for 10.9% and 5.6% of the total variance, respectively. The scree plot (Fig. 4c) showed that the first few principal components captured the majority of genetic variance, whereas subsequent components contributed progressively less variation, indicating clear population stratification.

The IBD-based hierarchical clustering and PCA revealed a geographic basis for genetic structure. South Asian accessions formed the largest genetic group (*N* = 232, 63.0%), primarily represented by Indian materials (*N* = 226, 61.4%), along with accessions from Pakistan (*N* = 5, 1.4%) and Sri Lanka (*N* = 1, 0.3%), reflecting the center of diversity and domestication for mung bean. East Asian accessions (*N* = 34, 9.2%), predominantly from Korea (*N* = 33, 9.0%), formed a distinct cluster, suggesting genetic differentiation associated with regional breeding and selection. Southeast Asian materials (*N* = 30, 8.2%), mainly from the Philippines (*N* = 23, 6.2%) and Thailand (*N* = 7, 1.9%), clustered separately, while accessions from West/Central Asia (*N* = 12, 3.3%) showed intermediate positioning. A notable proportion of accessions (*N* = 37, 10.1%) with unknown geographic origin exhibited admixed genetic backgrounds. The remaining accessions from Europe (*N* = 11, 3.0%), Oceania (*N* = 8, 2.2%), and other regions represented minor genetic groups.

### Phenotypic structure and PCA

**Fig. 5.**
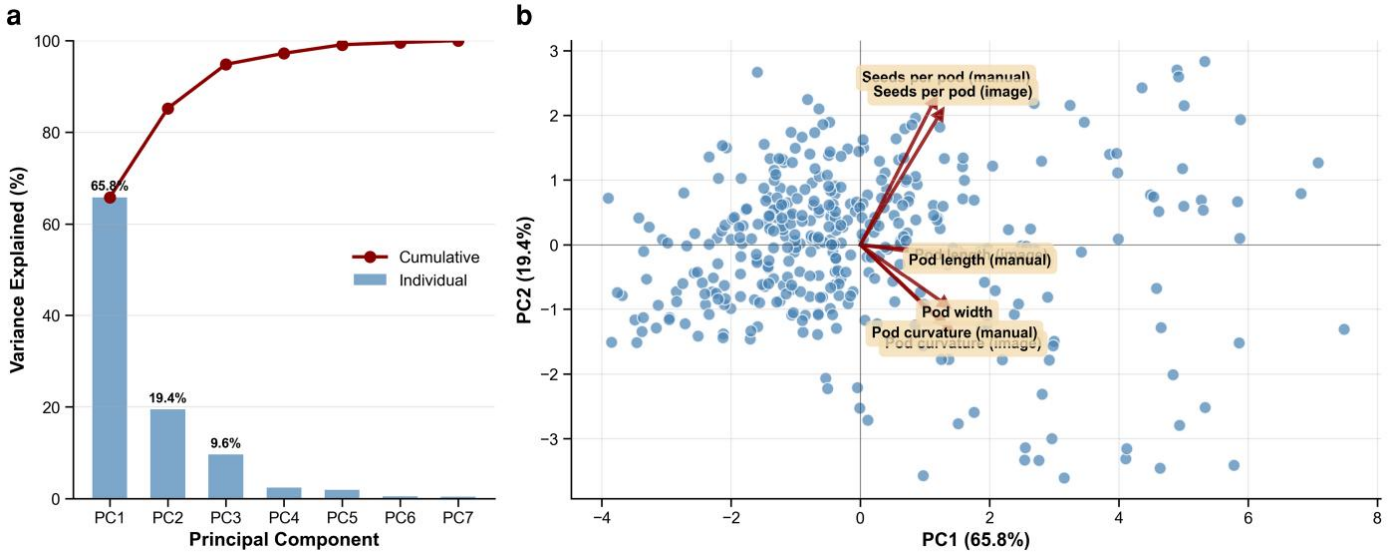
PCA based on BLUEs values from 372 genotypes across 7 morphological traits. a) Scree plot showing individual and cumulative variance explained by principal components. b) PCA biplot displaying genotype scores (points) and trait loading vectors (arrows) in PC1-PC2 space. PC1 (65.8% variance) represents overall pod morphology, while PC2 (19.4% variance) contrasts SPP with PC traits.

PCA indicated that 85.2% of the total phenotypic variation was captured by the first 2 components (Fig. 5). PC1 explained most of the variance (65.8%) and was defined by general pod dimensions, showing uniform positive loadings (>0.34) for PL, PW, and PC among both manual and image-based methods. This component effectively ordered genotypes by overall pod size and robustness. PC2 (19.4% of variance) captured a morphological contrast between seed potential and pod shape, driven by strong positive loadings for SPP (manual: 0.61; image: 0.56) and negative loadings for image-based PC (−0.37). Consequently, PC2 distinguishes genotypes that prioritize high seed number from those that exhibit pronounced PC. An additional 9.6% of the variance was explained by PC3, which primarily accounted for residual variation in PW.

### GWAS results

To unravel the genetic architecture underlying the observed phenotypic variation in pod morphological traits, GWAS was conducted using both manually measured and image-analysis-based predicted datasets.

### Comparison of manual and image-based phenotyping data in GWAS

**Fig. 6.**
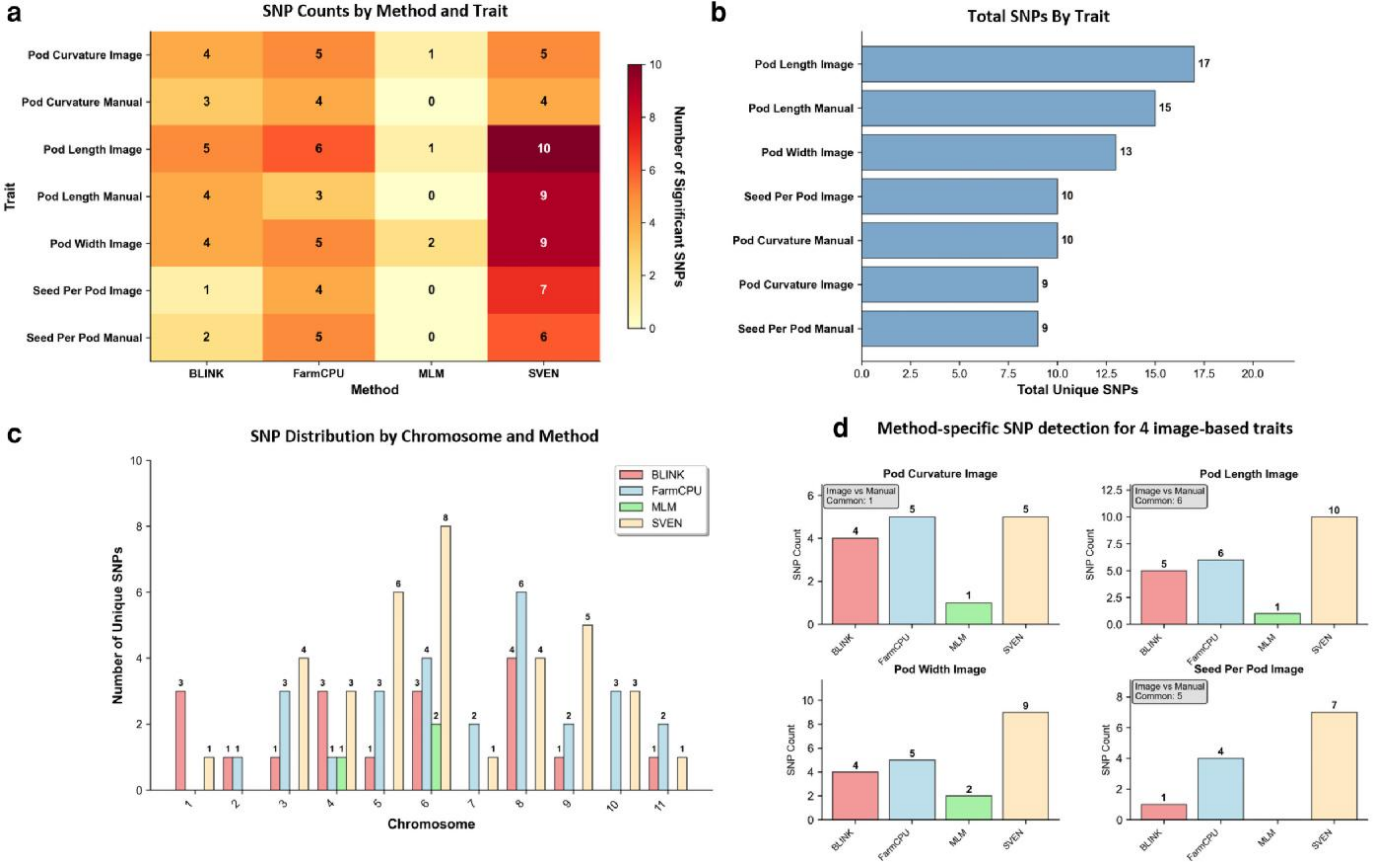
Comparative analysis of GWAS methods for pod and seed trait detection across multiple phenotyping approaches. a) Heatmap showing the number of significant SNPs detected by 4 GWAS methods (BLINK, FarmCPU, MLM, and SVEN) across pod morphological traits. b) Total unique SNPs identified per trait across all GWAS methods. c) Chromosomal distribution of significant SNPs by the GWAS method. The grouped bar chart displays the number of unique SNPs detected by each method on chromosomes 1–11. d) Method-specific SNP detection for 4 image-based traits.

To dissect the genetic architecture of pod morphology, GWAS was conducted using 4 statistical models (FarmCPU, BLINK, MLM, and SVEN). The analysis identified 49 significant, unique SNPs for image-derived traits, compared with 33 for manual measurements (Supplementary Table 4). The image-based platform demonstrated superior genomic resolution, particularly for complex traits. For PC, image-based GWAS detected 15 significant loci, whereas manual scoring identified only 11. Notably, the image-based analysis identified pleiotropic signals on Chromosomes 2 and 6 that were undetectable using manual visual scores (Fig. 6). Interestingly, all 4 models identified common signals on chromosomes 4 and 6 (Fig. 6c).

### Pleiotropic loci governing pod morphological traits

**Fig. 7.**
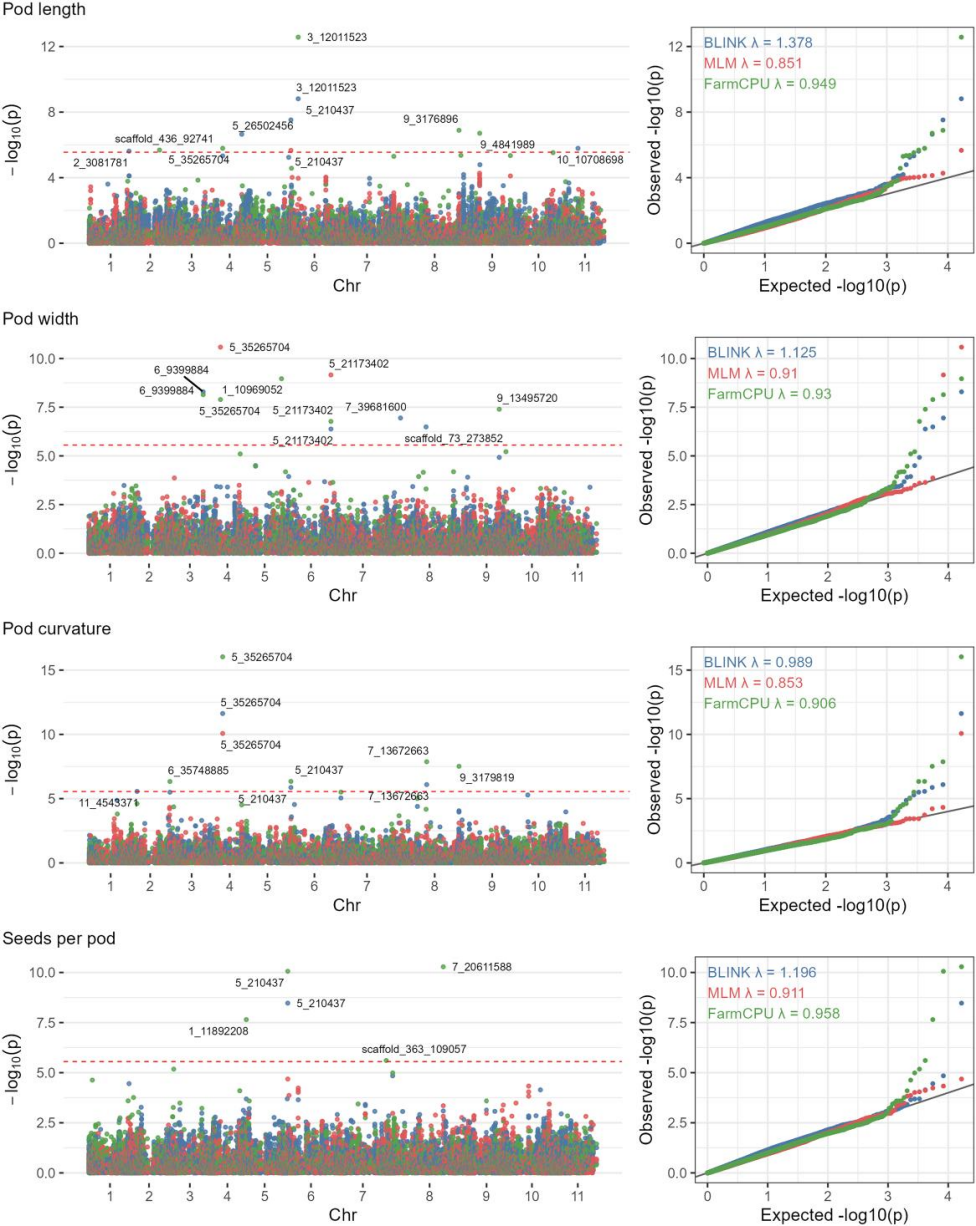
Manhattan and corresponding Q-Q plots that illustrate the results of genome-wide association studies (GWAS) based on the FarmCPU, BLINK, and MLM methods, focusing on image-based phenotypic traits, PL, PW, PC, and SPP. The dashed line in the Manhattan plots indicates the Bonferroni genome-wide correction threshold set at α = 0.05 (−log_10_(*P*) ≥ 5.56). The solid line in Q-Q plots represents the expected distribution of *P*-values under the null hypothesis of no association. Deviations above the dashed line indicate SNPs with stronger associations than expected by chance. Results from the SVEN method are not visualized in these plots, as SVEN outputs the proportion of variance explained (PVE) and posterior probabilities rather than the continuous *P*-values required for traditional Manhattan and Q-Q plots (see Table 2 and Supplementary Table 4 for SVEN results).

Two major genomic regions demonstrated significant pleiotropic effects across multiple pod traits (Fig. 7 and Table 2). SNP 5_35265704 on Chromosome 4 was the most significant association, co-localizing across 4 GWAS models for PL, PW, and PC. This SNP explained a larger proportion of phenotypic variance for image-based PC (30%) and PW (26.3%). Notably, while manual phenotyping detected this locus for PC using specific multilocus models (eg BLINK; *P* = 1.58 × 10^−15^), it failed to capture the association consistently across all methods (eg FarmCPU and MLM) and explained only 15.9% of the phenotypic variance. In contrast, the continuous image-based index identified the association robustly across all 4 tested models (MLM, FarmCPU, BLINK, and SVEN), achieving a higher level of significance (*P*_image_ = 9.27 × 10^−17^ in FarmCPU).

**Table 2.** Details of significant SNPs detected by 2 or more GWAS methods (MLM, BLINK, FarmCPU, and SVEN) for image-based pod morphological traits in mungbean.

Trait	SNP ID	Chr	Position (bp)	Ref/Alt	GWAS model	MAF	Allelic Effect	SE	*P*-value range	PVE (%)	Candidate gene	Protein name
PC	5_35265704	4	7227,756	T/C	BLINK, farm CPU, MLM, SVEN	0.09	0.03	0.00	9.27 × 10^−17^–8.5 × 10^−11^	30.0	Virad04G0076900	IAA-amido synthetase
	5_210437	6	279,187	T/G	BLINK, FarmCPU, SVEN	0.18	0.01	0.00	4.5 × 10^−7^–1.35 × 10^−6^	5.3	Virad06G0002400	Potassium transporter-like protein
	7_13672663	8	29,808,835	T/A	BLINK, FarmCPU	0.26	0.00	0.00	1.35 × 10^−8^–8.04 × 10^−7^	0.1	Virad08G0248300	IAA-amido synthetase
PL	3_12011523	6	7,320,433	G/A	BLINK, FarmCPU, SVEN	0.02	−1.33	0.11	2.70 × 10^−13^–1.54 × 10^−9^	27.1	Virad06G0073800	Alpha/beta-hydrolase superfamily protein
	5_210437	6	279,187	T/G	BLINK, MLM, SVEN	0.18	0.54	0.08	3.01 × 10^−8^–2.15 × 10^−6^	11.5	Virad06G0002400	Potassium transporter-like protein
	5_35265704	4	7,227,756	T/C	FarmCPU, SVEN	0.09	0.74	0.10	1.61 × 10^−6^–1.61 × 10^−6^	12.3	Virad04G0076900	IAA-amido synthetase GH3.5-like
Pod width	5_21173402	6	42,482,684	C/A	BLINK, FarmCPU, MLM, SVEN	0.05	0.37	0.04	6.97 × 10^−10^–4.18 × 10^−7^	20.1	Virad06G0253300	AP2-like ethylene-responsive transcription factor
	5_35265704	4	7,227,756	T/C	FarmCPU, MLM, SVEN	0.09	0.33	0.03	2.60 × 10^−11^–1.28 × 10^−8^	26.3	Virad04G0076900	IAA-amido synthetase GH3.5-like
	6_9399884	3	35,693,342	T/G	BLINK, FarmCPU	0.41	0.22	0.05	5.13 × 10^−9^–7.21 × 10^−9^	5.9	Virad03G0209100	WRKY transcription factor
	9_13495720	9	30,855,923	C/T	FarmCPU, SVEN	0.50	0.14	0.02	4.02 × 10^−8^–4.02 × 10^−8^	13.0	Virad09G0151900	RING/U-box superfamily protein
SPP	5_210437	6	279,187	T/G	BLINK, FarmCPU, SVEN	0.18	0.53	0.06	8.72 × 10^−11^–3.38 × 10^−9^	15.9	Virad06G0002400	Potassium transporter-like protein
	7_49541747	8	1,427,958	C/T	BLINK, FarmCPU, SVEN	0.06	0.54	0.08	2.80 × 10^−7^–1.92 × 10^−6^	11.4	Virad08G0016500	Alpha/beta superfamily hydrolase

Ref/Alt indicates the reference and alternate alleles. GWAS Methods lists which GWAS models identified the SNP. Allelic Effect represents the mean effect size, and SE represents the standard error across methods that provided effect estimates. *P*-value Range shows the minimum and maximum *P*-values across methods. PVE (%) indicates the percentage of PVE, showing mean and range when multiple estimates were available. MAF is the minor allele frequency. Significance threshold: −log_10_(*P*) ≥ 5.56 (Bonferroni correction: α = 0.05/17,928 SNPs).

Chromosome (Chr) and position are based on the recently published mungbean reference “*Weilv*-9' genome, *vigra.Weilv-9.gnm1.ann1* (Jia et al. 2025) at the *Legume Information System* website. (https://data.legumeinfo.org/Vigna/radiata/genomes/Weilv-9.gnm1.5TZZ/).

Another pleiotropic SNP, 5_210437 on Chromosome 6, was found to be associated with PL, PC, and SPP across multiple models (FarmCPU, BLINK, MLM, and SVEN), with PVE values ranging from 5.27% to 15.86%. While the association was highly significant for SPP in both image-based (*P* = 8.7 × 10^−11^) and manual (*P* = 1.8 × 10^−7^) datasets, the genetic signal for geometric traits was primarily captured by the imaging platform. This SNP explained 15.9% of the variance in image-based SPP, compared with 12.4% for the manual method. More notably, for PC, the association was successfully identified by FarmCPU and SVEN in the image-based data but was completely undetectable in the manual phenotypic data. Similarly, for PL, the signal was supported by 3 models (BLINK, MLM, and SVEN) in the image-based analysis, but only the SVEN model was detected using manual measurements (Supplementary Fig. 1).

### Trait-specific associations

Beyond the pleiotropic regions, trait-specific associations provided further insights into pod architecture (Fig. 7; Supplementary Table 4). For the PC, image-based GWAS identified unique associations on Chromosome 3 (6_35748885; PVE = 20%) and Chromosome 8 (7_13672663; PVE = 0.1%). For PL, of the 17 SNPs identified, SNP 3_12011523 on Chromosome 6 was identified by BLINK, FarmCPU, and SVEN. A total of 13 significant SNPs were identified for PW, distributed across 7 chromosomes. Notably, a strong signal on Chromosome 6 (5_21173402) was consistently identified by all 4 models, accounting for 20% of the phenotypic variation, apart from the major locus on Chromosome 4 (5_35265704). In addition, SNP 6_9399884 (Chr 3) and SNP 9_13495720 (Chr 9) were collectively identified by FarmCPU and SVEN models for pod width. Finally, for SPP, among 10 significant SNPs, apart from the shared Chromosome 6 locus (5_210437), significant signals were found on Chromosome 5 (1_11367629, PVE = 13.6%), and Chromosome 8 (7_49541747, PVE = 12%).

### Cross-species synteny and candidate gene identification

To validate the genomic position of the major GWAS signals, comparative mapping was performed against *Vigna unguiculata*, *Phaseolus vulgaris*, *Glycine max*, and *A*. *thaliana* (Supplementary Fig. 2). The pleiotropic SNP 5_35265704 is located on chromosome 4 at 7,227,756 base pairs. Within a 223 kb search radius of this marker (based on the genome-wide LD decay average at *r*^2^ = 0.2), we identified 25 annotated genes. Based on the gene functional description, *Virad04G0076900* was identified as a candidate gene. This gene is annotated as an indole-3-acetic acid (IAA)-amido synthetase. Synteny analysis indicates that the gene is orthologous to IAA-amido synthetase GH3 family genes *Glyma.12G103500 and Glyma.06G301000* in soybean and *Vignaun05g223100* in cowpea. The *Arabidopsis* orthologs, *GH3.17* (*AT2G47750*) and *GH3.9* (*AT4G27260*), are annotated in the TAIR database as auxin-responsive genes involved in hypocotyl elongation and pod development.

For the Chromosome 2 locus (SNP 5_210437), a cluster of potassium transporter genes falls within a 36.5 kb window. Gene, *Virad06G0002400* was identified as a candidate based on its gene functional description. This gene has high sequence identity with potassium transporter orthologs in cowpea (*Vigun09g000800*) and common bean (*Phvul.009G262700*), and co-orthologs *Glyma.03G264100* and *Glyma.19G263100* in soybean. This gene shares conserved synteny with the *Arabidopsis* gene *HAK5* (*AT4G13420*), which encodes a high-affinity potassium transporter expressed in root and reproductive tissues.

### Genomic prediction

#### GP accuracy

**Fig. 8.**
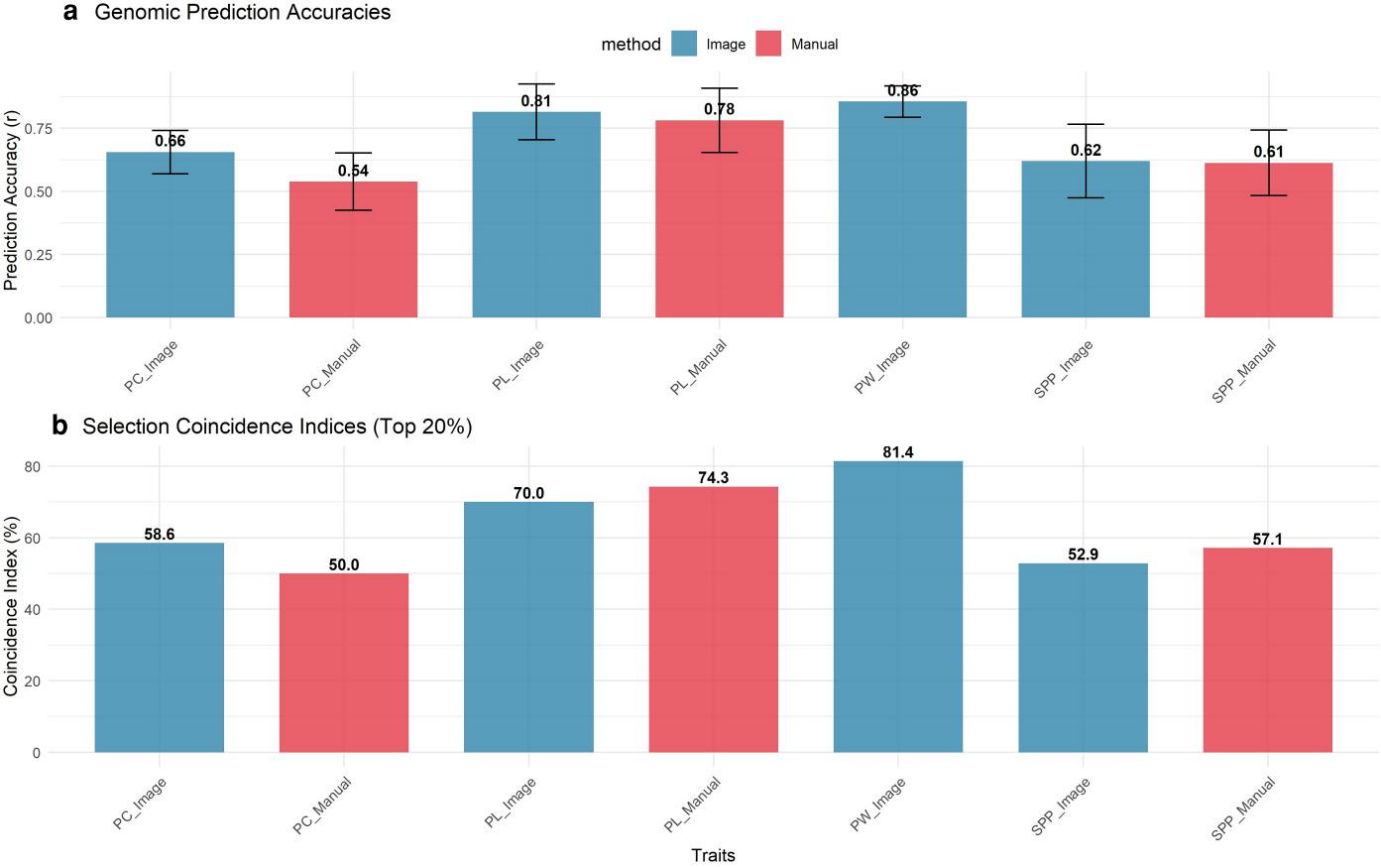
GP accuracies and selection coincidence indices of mungbean pod morphological traits. a) GP accuracies (correlation coefficients, *r*) between observed trait BLUPs and GEBVs from 10-fold CV using rrBLUP. Error bars represent standard deviation across CV folds. b) Selection coincidence indices between top 20 genotypes selected based on BLUPs vs GEBVs for each trait. Numbers on bars indicate coincidence index values, while numbers in parentheses show the count of common genotypes out of top 20 selections.

GPA estimated via 10-fold CV varied across traits, consistent with the broad-sense heritability estimates (*H*^2^) (Fig. 8; Supplementary Table 7). Image-based PW exhibited the highest prediction accuracy (*r* = 0.86 ± 0.06). For traits measured by both methods, image-based phenotyping consistently yielded accuracy comparable to or higher than that of manual measurements. The most significant gain was observed for PC, where the image-based index achieved an accuracy of *r* = 0.66 ± 0.09, representing a 22% improvement over the manual score (*r* = 0.54 ± 0.11). This suggests that the digital quantification of curvature captures genetic variation more precisely than subjective visual scoring. Image-based PL also demonstrated high predictability (*r* = 0.8 ± 0.11), comparable to manual measurements (*r* = 0.78 ± 0.13). For SPP, prediction accuracies were moderate and nearly identical between methods (Image: *r* = 0.62 ± 0.15; Manual: *r* = 0.61 ± 0.13), consistent with the complex genetic architecture of this yield-component trait.

#### Selection coincidence index

To assess the practical utility of GEBVs, we calculated the SCI to determine the overlap between the top 20% of genotypes selected by phenotypic values (BLUPs) vs those selected by GEBVs. High coincidence was observed for PW, with an SCI of 81.4%. PL also showed strong selection correspondence, with SCI values of 70.0% for the image-based method and 74.3% for manual measurements. The coincidence for PC was moderate to high, with the image-based method (58.6%) outperforming manual scoring (50.0%).

## Discussion

This study demonstrates the effectiveness of image-based high-throughput phenotyping in capturing subtle and complex variations in pod morphological traits of mungbean (*V*. *radiata* (L.) R. Wilczek), a crop of growing importance for sustainable protein production. By integrating deep learning-based image segmentation with genome-wide association and prediction models, we gained valuable insights into the genetic architecture of pod morphological traits. Such knowledge can play a key role in accelerating genetic improvement and cultivar development in mungbean. The use of computer vision and machine learning has already transformed plant phenotyping by enabling high-throughput and objective measurement of complex traits (Singh et al. 2016; Singh et al. 2018; Parmley et al. 2019; Singh et al. 2021). To our knowledge, however, no previous reports have focused on image-based phenotyping of mung bean pod morphological traits.

In this study, the Epson Expression 11000XL scanner was used to obtain high-throughput imaging of mungbean pods at 300 dpi. Previous studies have demonstrated that this scanner is effective in seed and root phenotyping, showcasing its reliability in capturing detailed morphological features (Pace et al. 2014; Zhu et al. 2021; de Carvalho Miranda et al. 2023; Klasen et al. 2025). Our work, however, expands its application to include pod morphological traits, which involves a destructive phenotyping method. The strong correlation between image-based and manual phenotyping is particularly evident for PL (*r* = 0.96) and SPP (*r* = 0.96), highlighting the reliability of image-analysis-based trait extraction. This reliability extends to complex shape traits, where the image-derived curvature index demonstrated a robust correlation (*r* = 0.82) with manual visual scores. This high concordance validates the digital metric against the standard breeding method, confirming that the platform captures the biological ground truth. Beyond mere alignment, the imaging pipeline offers a distinct advantage by quantifying traits such as PC and PW, which are laborious and subjective to measure manually, on a continuous scale. This resolves phenotypic variation that is typically compressed by discrete visual scoring. By employing DeepLabV3 with a ResNet34 backbone, we achieved robust object segmentation and precise trait measurement, reducing rater bias and increasing throughput. Specifically, while manual measurement of PL and SPP typically requires 7 to 10 min of active labor per genotype, our digital pipeline requires only 2 min for image acquisition (scanning) followed by automated computational processing. This capability is especially important for breeding programs that manage large diversity panels, as well as for phenotyping advanced breeding lines to select potential high-yielding cultivars based on data-driven criteria, where consistency and accuracy in trait measurement are crucial.

The results reveal extensive phenotypic diversity and strong genetic control for most pod traits in mungbean, supporting their potential as selection targets in breeding programs. The observed correlations among pod dimensions further indicate an opportunity to improve yield components in a coordinated manner, while the comparatively lower correlation between SPP and pod dimensions suggests opportunities for independent selection to tailor trait combinations. The high heritability (0.74–0.91) of these pod morphological traits in mungbean makes them strong candidates for genome-assisted breeding. The GWAS conducted using 4 statistical models (FarmCPU, BLINK, MLM, and SVEN) identified 109 significant associations, corresponding to 65 unique SNPs across the genome. Among these, 2 loci were associated with multiple pod morphological traits, suggesting significant pleiotropy and warranting further investigation. Crucially, the high co-localization observed between the multilocus models (FarmCPU, BLINK, and SVEN) validates these signals, mitigating the risk of false positives often associated with high-throughput phenotypic data. Comparative mapping, supported by GWAS results, allowed us to identify potential candidate genes at each locus on chromosomes 4 and 6. SNP 5_35265704 is particularly noteworthy as it is consistently associated with PL, PW, and PC. A plausible candidate gene, Virad04G0076900, was identified 15.6 kb from the significant SNP. This gene is a member of the auxin-responsive GH3 family and is orthologous to At4g27260 (GH3.5) in *A*. *thaliana* (Luo et al. 2023). This gene has been shown to be linked to auxin homeostasis and organ elongation (Staswick et al. 2005; Park et al. 2007). The *Virad04G0076900* protein shares 92.6% amino acid identity with the cowpea (*V*. *unguiculata*) ortholog *Vigun05g223100*, which resides within the primary region on linkage group 1 associated with PL (Xu et al. 2017). This syntenic correspondence suggests that the mungbean chromosome 1 locus represents an evolutionarily conserved genomic region controlling pod elongation across *Vigna* species, reinforcing the role of *GH3*-mediated auxin regulation in shaping pod morphology. Our findings reinforce earlier reports that highlight the importance of *GH3*-mediated auxin conjugation in regulating pod development and curvature in both legumes (Song et al. 2016; Lo et al. 2018; Lv et al. 2023) and *Arabidopsis*.

A gene, *Virad06G0002400*, located 35.6 kb from SNP 5_210437, is associated with both PL and the SPP. This gene encodes a potassium transporter in the HAK5 family, and its *Arabidopsis* ortholog is *AT4G13420* (Maierhofer et al. 2024). This pleiotropic association aligns perfectly with the dual physiological roles of potassium (*K*^+^) in plant reproductive development (Nieves-Cordones and Rubio 2025). This finding suggests a functional link between pod elongation and the number of SPP in mung bean plants, and potential pleiotropy of this locus for these 2 traits. Additionally, the identification of overlapping locations for PL, PW, PC, and SPP suggests shared genetic regulation, enabling simultaneous marker-assisted selection of multiple traits.

Our GP results demonstrate that image-based phenotyping is not only a high-throughput alternative to traditional manual assessment but also often a superior method for capturing heritable variation in legume pod morphology. The consistently high prediction accuracies (*r* > 0.60 for most trait-method combinations) indicate significant potential for implementing genomic selection in mungbean breeding programs. Both PL and PW demonstrated the highest prediction accuracies (*r* > 0.80), corresponding to traits with significant genetic control, enabling highly effective genomic selection strategies. Notably, PW (measured only via imaging) achieved the highest selection coincidence of 0.81, meaning that genomic models could successfully identify most top-performing genotypes without field phenotypic selection. A key finding of this study is the demonstrated superiority of image-derived phenotypes for complex shape traits. This precision translates into a better ranking of elite genotypes, as evidenced by the higher coincidence index. Consequently, image-based phenotyping for curvature serves a dual role: it enhances genetic resolution for QTL discovery (as seen in GWAS) and improves the reliability of individual selection in breeding pipelines. For count-based traits like SPP, which exhibited moderate heritability, both methods yielded comparable intermediate predictive performance (*r* ≈ 0.61). Manual phenotyping showed a marginally higher selection coincidence (0.57 vs 0.53), suggesting that direct visual counting remains highly effective for discrete traits. The lower selection coincidence observed for SPP compared to structural traits, such as PW, is consistent with the complex genetic architecture of yield components. While PW is a relatively stable morphological trait, final seed number is highly polygenic and typically more sensitive to environmental variations during development. However, for continuous geometric traits, especially those difficult to quantify manually, such as curvature, computer vision offers a clear advantage for quantifying heritable genetic signals. These findings align with recent reports in other legumes and cereal crops, where high-quality automated phenotyping has improved GP accuracy compared to traditional manual measurements (Li et al. 2018; Azodi et al. 2019; Crossa et al. 2025). This supports the growing consensus that phenotyping quality, rather than just genotyping density is a critical factor limiting the speed of breeding advancements (Araus et al. 2018).

## Conclusions

The findings from this study can benefit the global mungbean crop improvement community, particularly in emerging cultivated regions such as the Midwest, where a growing demand for plant-based protein is driving development. Genomic tools combined with high-throughput image-based phenotyping, help breeders efficiently test large populations, making the process less resource-intensive while maintaining high accuracy. Identifying true trait-associated loci and predictive markers is essential for developing effective and accurate marker-assisted selection and genomic selection strategies, which positively contribute to yield-related traits (Xiao et al. 2022; Pinto et al. 2023). In addition, genetic analysis of pod morphological traits often associated with harvest index and consumer preferences, can help trait pyramid approaches. This ultimately strengthens breeders to simultaneously improve both productivity and market appeal (García-Fernández et al. 2024).

While our scanner-based imaging method has proven effective, it restricts our ability to conduct large-scale studies on trait development across different growth stages. Future research should focus on nondestructive phenotyping platforms, such as drone or rover-mounted imaging systems, which can capture pod traits over time without the need for manual harvesting. These technologies would allow us to create larger, more precise datasets while reducing the resources required for genetic analysis studies. Additionally, although our GP models have achieved moderate to high accuracy, there is still room for improvement to ensure more reliable selection decisions. The predictive power and robustness of genomic selection pipelines could be enhanced by integrating data from multiple environments, expanding the diversity of the training population, and incorporating multiomics data.

This research demonstrates the transformative potential of combining image-based phenotyping with genomic tools in mungbean breeding. Our integrated approach enhances phenotyping accuracy, improves prediction power, and accelerates the discovery of functional genetic variants. As breeding programs increasingly adopt automated and precision techniques, such integrative approaches will be crucial for developing high-yielding, climate-resilient legume cultivars.

## Supplementary Material

jkag106_Supplementary_Data

## Data Availability

Raw phenotypic data, including both image-based and manual measurements, BLUEs, BLUPs of these traits, and Genomic prediction results, are provided as supplementary files. The mungbean genotypic dataset utilized in this study is publicly accessible through the Legume Information System (https://data.legumeinfo.org/Vigna/radiata/genomes/Weilv-9.gnm1.5TZZ/). All analysis scripts used in this study are publicly available at GitHub: https://github.com/vboddepalli89/Image-based-pod-phenotyping. Supplemental material available at G3 online.
