# Supplementary material for: Image-based high-throughput phenotyping enables genetic analyses of pod morphological traits in mungbean (Vigna radiata (L.) R. Wilczek)
Source: G3 (Bethesda). 2026 Apr 28;16(6):jkag106. doi: 10.1093/g3journal/jkag106 (PMC13233092; doi:10.1093/g3journal/jkag106)
Supplement: jkag106_Supplementary_Data [file jkag106_supplementary_data.zip › Supplemental_Figure_Legends_G3-2026-406606.docx]

**SUPPLEMENTARY FIGURE LEGENDS**

**Supplementary Figure 1:** Manhattan and corresponding Q-Q plots that illustrate the results of genome-wide association studies (GWAS) based on the MLM, FarmCPU, and BLINK methods, focusing on manual-based phenotypic traits, PL, PC, and SPP. The red dashed line in the Manhattan plots indicates the Bonferroni genome-wide correction threshold set at α = 0.05 (-log₁₀(P) ≥ 5.56). The black line in Q-Q plots represents the expected distribution of p-values under the null hypothesis of no association. Deviations above the red line indicate SNPs with stronger associations than expected by chance.

**Supplementary Figure 2**: Comparative homology and tissue-specific expression of GH3 and HAK5 family genes (A, B). Chord diagrams illustrate the BLASTp-based homology of mung bean GH3 candidate proteins with orthologs from Glycine max (gly), Phaseolus vulgaris (pha), adzuki bean (*Vigna angularis*) and *Vigna unguiculata* (vig). Panel A corresponds to the GH3.5-like gene (*Virad04G0076900*), while panel B corresponds to another HAK5 candidate (*Virad06G0002400*). The thickness of the ribbons indicates the strength of the alignment and the level of sequence similarity, highlighting gene conservation across species. (C). Tissue-specific expression profiles of Arabidopsis GH3.5 (*AT4G27260*, WES1) and (D) Tissue-specific expression profiles of Arabidopsis HAK5 (*AT4G13420*) retrieved from AtGenExpress eFP browser (Klepikova et al. 2016). Expression is visualized across various developmental stages and organs, with red indicating high expression and yellow indicating moderate expression
