# Supplementary figures and images for: Image-based high-throughput phenotyping enables genetic analyses of pod morphological traits in mungbean (Vigna radiata (L.) R. Wilczek)
Source: G3 (Bethesda). 2026 Apr 28;16(6):jkag106. doi: 10.1093/g3journal/jkag106 (PMC13233092; doi:10.1093/g3journal/jkag106)

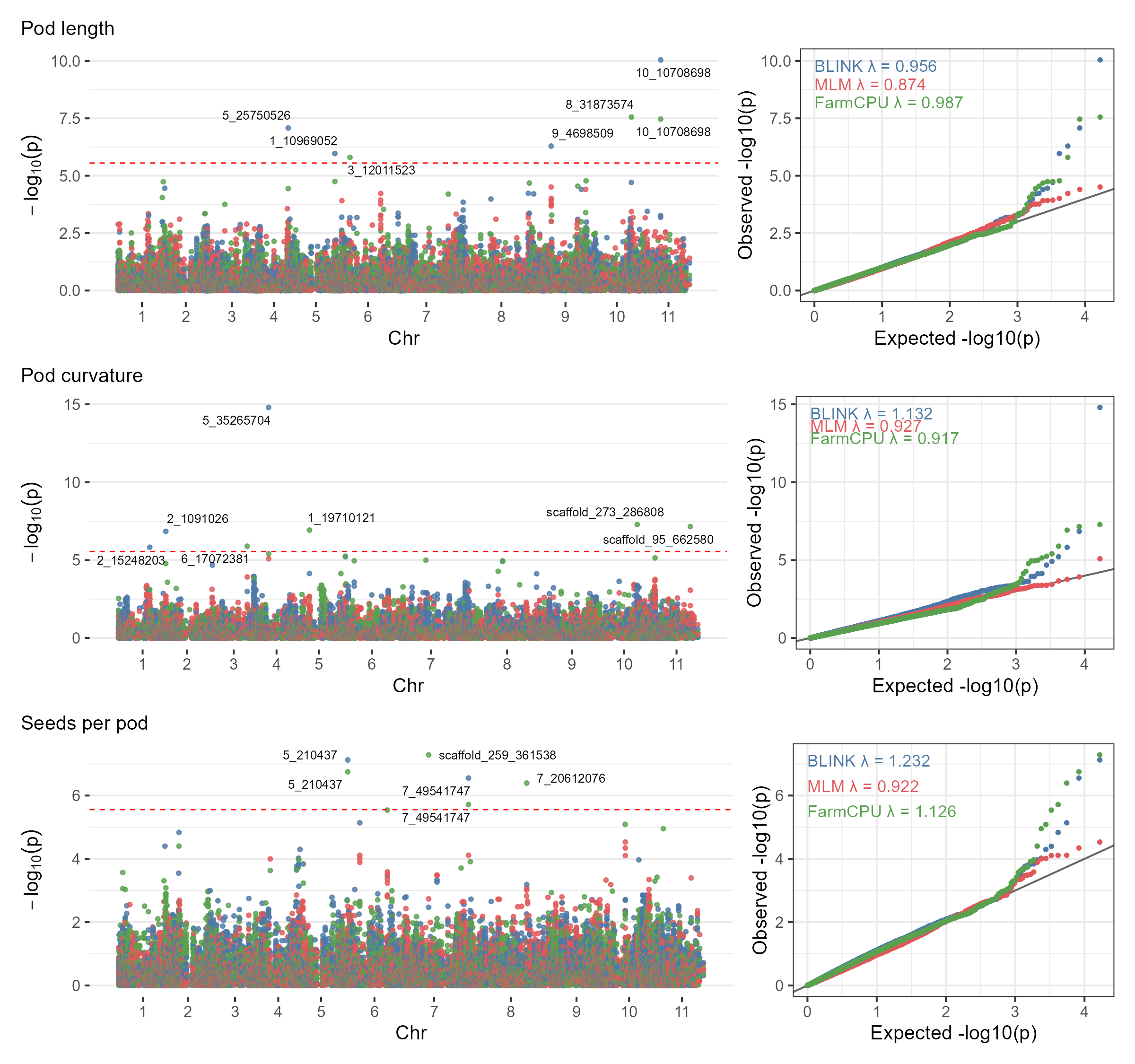

Supplement: jkag106_Supplementary_Data [file jkag106_supplementary_data.zip › Supplemental_Figure_1_G3-2026-406606.png]

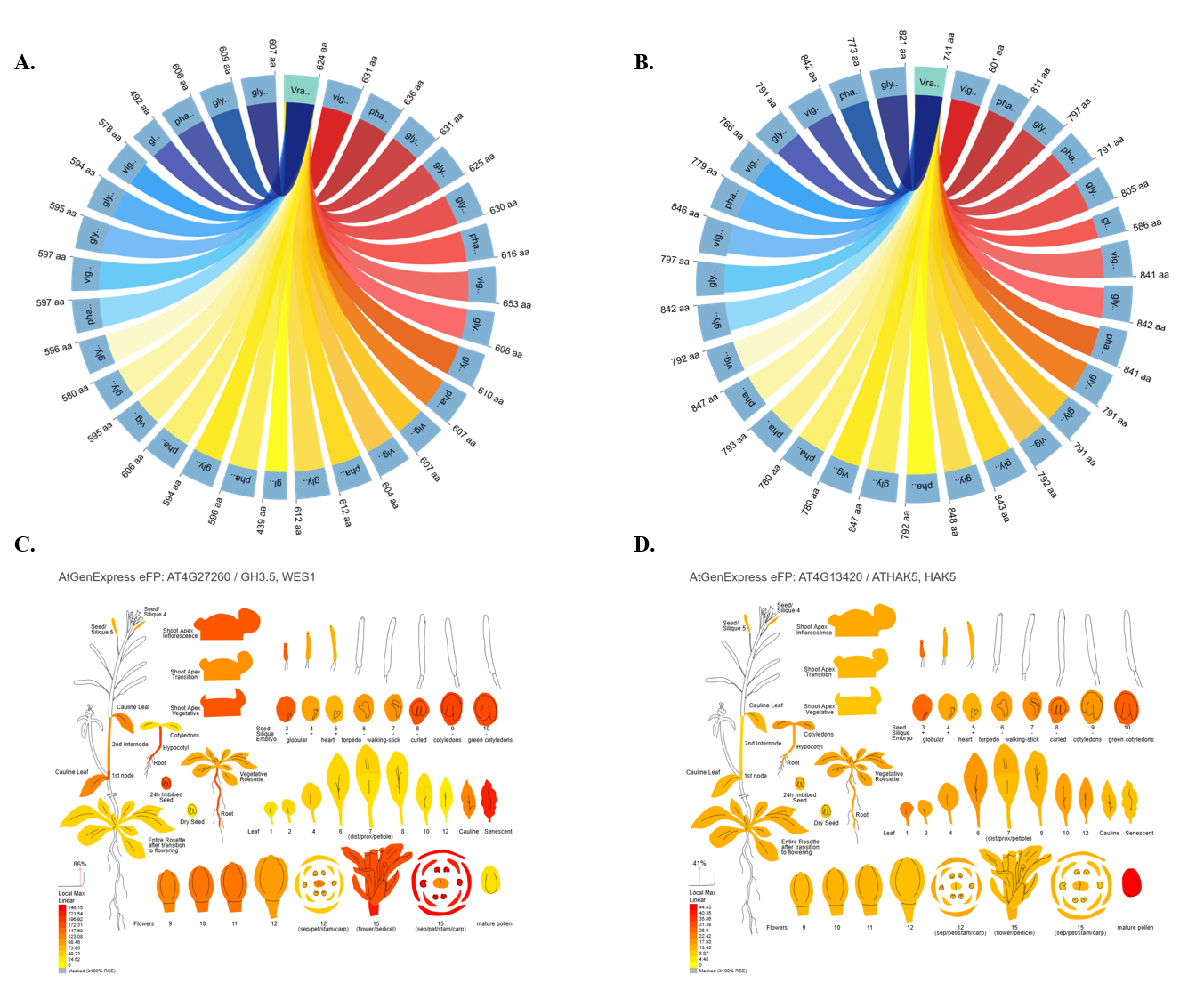

Supplement: jkag106_Supplementary_Data [file jkag106_supplementary_data.zip › Supplemental_Figure_2_G3-2026-406606.png]
